# Benign gastric outlet obstruction: evolving strategies from surgery to endoscopic ultrasound-guided gastrojejunostomy

**DOI:** 10.1177/26317745251398950

**Published:** 2026-03-03

**Authors:** Giacomo Emanuele Maria Rizzo, Giuseppe Infantino, Gabriele Rancatore, Dario Quintini, Dario Ligresti, Nicoletta Belluardo, Giuseppe Rizzo, Elio D’amore, Marco Giacchetto, Ilaria Tarantino

**Affiliations:** Department of Diagnostic and Therapeutic Services, Gastroenterology and Endoscopy Unit, Istituto Mediterraneo dei Trapianti e Terapie ad Alta Specializzazione, ISMETT-IRCCS, Palermo, Italy; Department of Diagnostic and Therapeutic Services, Endoscopy Service, IRCCS – ISMETT, Via Tricomi 5, 90127 Palermo, Italy; Department of Diagnostic and Therapeutic Services, Gastroenterology and Endoscopy Unit, Istituto Mediterraneo dei Trapianti e Terapie ad Alta Specializzazione, ISMETT-IRCCS, Palermo, Italy; Department of Diagnostic and Therapeutic Services, Gastroenterology and Endoscopy Unit, Istituto Mediterraneo dei Trapianti e Terapie ad Alta Specializzazione, ISMETT-IRCCS, Palermo, Italy; Department of Diagnostic and Therapeutic Services, Gastroenterology and Endoscopy Unit, Istituto Mediterraneo dei Trapianti e Terapie ad Alta Specializzazione, ISMETT-IRCCS, Palermo, Italy; Department of Diagnostic and Therapeutic Services, Gastroenterology and Endoscopy Unit, Istituto Mediterraneo dei Trapianti e Terapie ad Alta Specializzazione, ISMETT-IRCCS, Palermo, Italy; Department of Diagnostic and Therapeutic Services, Gastroenterology and Endoscopy Unit, Istituto Mediterraneo dei Trapianti e Terapie ad Alta Specializzazione, ISMETT-IRCCS, Palermo, Italy; Department of Diagnostic and Therapeutic Services, Gastroenterology and Endoscopy Unit, Istituto Mediterraneo dei Trapianti e Terapie ad Alta Specializzazione, ISMETT-IRCCS, Palermo, Italy; Department of Diagnostic and Therapeutic Services, Gastroenterology and Endoscopy Unit, Istituto Mediterraneo dei Trapianti e Terapie ad Alta Specializzazione, ISMETT-IRCCS, Palermo, Italy; Department of Diagnostic and Therapeutic Services, Gastroenterology and Endoscopy Unit, Istituto Mediterraneo dei Trapianti e Terapie ad Alta Specializzazione, ISMETT-IRCCS, Palermo, Italy

**Keywords:** benign, bGOO, endoscopic ultrasound, endoscopy, EUS, EUS-GJ, gastric outlet obstruction, gastroenterostomy, gastrojejunostomy, GOO

## Abstract

Benign gastric outlet obstruction (bGOO) presents a significant therapeutic challenge, with etiologies ranging from peptic strictures to complex postsurgical or inflammatory conditions. While surgery has historically offered durable outcomes, its morbidity in frail populations underscores the need for effective, less invasive alternatives. This review critically examines the current literature up to April 2025 on surgical, endoscopic, and endoscopic ultrasound-guided interventions for bGOO, evaluating technical success, clinical outcomes, recurrence rates, and adverse events. Endoscopic balloon dilation shows excellent efficacy in simple peptic strictures but has limited efficacy in anatomically complex cases. Fully covered self-expandable metal stents can provide temporary relief but are associated with significant migration risk. Among emerging techniques, endoscopic ultrasound-guided gastrojejunostomy (EUS-GJ) stands out by combining the anatomical efficacy of surgery with the minimal invasiveness of endoscopy. Recent studies report technical and clinical success rates exceeding 95%, with lower recurrence and complication rates compared to traditional approaches. Comparative data increasingly support EUS-GJ as the preferred option in refractory or high-risk patients. Tailoring treatment strategies based on etiology, anatomical complexity, and patient condition is essential. EUS-GJ is redefining the therapeutic landscape of bGOO, offering a minimally invasive and durable alternative to surgery in carefully selected cases.

## Plain language summary

Novel therapeutic approaches improve outcomes for patients with obstructive gastrointestinal symptoms caused by benign diseases

Some people may develop a gastrointestinal obstruction at the exit of the stomach, caused by scarring, inflammation, or previous surgery. This condition, known as benign gastric outlet obstruction (bGOO), can lead to symptoms such as nausea, vomiting, weight loss, and malnutrition. While surgery has long been the standard treatment, it may not be suitable for elderly or frail patients due to its associated risks. This review examines more modern and less invasive therapies, including an innovative technique called endoscopic ultrasound-guided gastrojejunostomy (EUS-GJ), which creates a new pathway for food to bypass the obstruction. Recent studies show that EUS-GJ is highly effective, safe, and associated with faster recovery times. It may soon become the first-line treatment for selected patients with benign gastric outlet obstruction.

## Introduction

Gastric outlet obstruction (GOO) is a condition characterized by mechanical impaired gastric emptying, leading to nausea, vomiting, weight loss, and poor oral intake. While malignancies are the most common cause, a minority of cases are due to benign conditions such as peptic ulcer-related stenosis, chronic and acute pancreatitis, caustic ingestion, postoperative adhesions, Crohn’s disease, superior mesenteric artery (SMA) syndrome, and median arcuate ligament syndrome (MALS).^1^ Historically, surgical gastrojejunostomy (SGJ) has been considered the gold standard for the management of benign GOO (bGOO), providing effective and long-lasting symptom relief.^2^ However, surgery carries potential risks such as infection, anastomotic leak, delayed gastric emptying, and prolonged recovery times, particularly in elderly or frail patients. Moreover, the technical demands of surgery and the need for general anesthesia may limit its feasibility in patients with multiple comorbidities.^3^ In recent years, advances in endoscopic techniques have led to less invasive approaches, such as endoscopic ultrasound-guided gastrojejunostomy (EUS-GJ). This technique offers several advantages, including reduced invasiveness, shorter hospital stays, and high technical success rates.^4^ Nonetheless, questions remain regarding its long-term effectiveness, complication rates, and optimal patient selection criteria, particularly in benign conditions, where evidence is limited and the optimal therapeutic algorithm remains uncertain.^5,6^ The aim of this review is to provide a comprehensive and updated overview of current treatment strategies for bGOO, comparing the outcomes, advantages, and limitations of both surgical and endoscopic approaches, and discussing emerging indications for EUS-GJ.

## Methods

A comprehensive search of the literature was conducted up to April 2025 into the major databases (Embase, Medline via PubMed, Scopus, and Cochrane Library). We included all relevant information regarding the treatment of bGOO, with a particular focus on surgical and endoscopic treatments. Search terms included keywords such as “benign gastric outlet obstruction,” “peptic stricture,” “endoscopic dilation,” “self-expandable metal stent,” “EUS-guided gastrojejunostomy,” and “surgical gastrojejunostomy.” Studies were screened and included if they (1) addressed the diagnosis, management, and outcomes of patients with benign GOO; (2) had particular attention to surgical and endoscopic techniques (including EUS-GJ, enteral stenting (ES), and endoscopic balloon dilation (EBD)); (3) included both populations (malignant and benign) when they were comparative studies; (4) in case of subgroup analyses for benign etiologies, data were available or could be reasonably extractable. Therefore, since many studies involve both benign and malignant GOO, particular attention was paid to distinguishing between the two groups to ensure a specific analysis of benign cases. Studies were excluded if they were not published in English or if they were on animals. Data were analyzed descriptively, and clinical success, complication rates, and recurrences were synthesized when available. Additionally, the influence of various factors on the results, such as patient characteristics, treatment type, and follow-up duration, was explored.

## Etiology of bGOO

Historically, peptic ulcer-related stenosis was one of the predominant causes of bGOO. This condition often represented a late-stage complication of chronic gastric or duodenal ulcers, occurring in a clinical context where *Helicobacter pylori* eradication was not systematic and pharmacological acid suppression was limited. The widespread introduction of proton pump inhibitors (PPIs), together with effective *H. pylori* eradication programs, has led to a substantial reduction in the incidence of peptic-related GOO.^3^ Chronic pancreatitis has become a more frequent cause due to the progressive fibrotic changes and duodenal compression it induces, similar to paraduodenal (groove) pancreatitis.^7,8^ Caustic injury from acid or alkali ingestion is a significant cause of bGOO due to scarring of the antrum or pylorus. The incidence of obstruction in this context ranges from 20% to 60%. In a study of 41 acid ingestion cases, 44.4% developed GOO, while another study found that 36.8% of 31 patients with alkali ingestion developed the condition.^9–11^ Chronic nonsteroidal anti-inflammatory drug (NSAID) use is another known etiology of GOO: these drugs promote pyloric edema and fibrosis, impair motility, and increase gastric secretion. In a study of 10 patients with NSAID-induced GOO, the duodenum was the most frequently involved site, followed by the combined duodenum and pylorus, and then the pylorus alone. Most strictures were short and web-like, and EBD was successful in 90% of cases.^12^ Additional causes, such as postsurgical anastomotic strictures, superior mesenteric artery syndrome, groove pancreatitis, and acute pancreatitis, may also lead to obstruction, particularly in the context of severe edema or inflammatory fluid collections. Less frequent etiologies include Crohn’s disease, pancreatic pseudocysts, radiation-induced strictures, post-inflammatory adhesions, inflammatory pseudotumor, gallbladder empyema, eosinophilic gastroenteritis (EoGE), and gastrointestinal tuberculosis.^13^ These findings confirm the heterogeneity of bGOO and highlight the importance of an accurate etiological diagnosis, which can be clinically useful to guide therapeutic decision-making. However, irrespective of the etiology, the ultimate goal of therapy is the resumption or improvement in oral intake, which can be graded using the gastric outlet obstruction scoring system (GOOSS)^14^ (Table 1).

**Table 1.** Gastric outlet obstruction scoring system.^14^

	GOOSS score
No oral intake	0
Liquids only	1
Soft solids	2
Low-residue or full diet	3

GOOSS, gastric outlet obstruction scoring system.

### Surgical treatment of bGOO

Surgery has long represented the definitive treatment for bGOO, and for many years was considered the therapeutic gold standard in the absence of minimally invasive alternatives. Although the advent of endoscopic techniques has progressively reduced the need for surgery in selected subgroups, it continues to play a central role in complex or refractory cases. Classical indications for surgery include failure of endoscopic therapy, non-dilatable or anatomically complex strictures, the presence of dense postoperative adhesions, and symptomatic recurrences in young or otherwise fit patients.^13^ The most commonly performed procedure is gastrojejunostomy (GJ), which consists of creating an anastomosis between the gastric wall and the jejunum to bypass the site of obstruction. This can be performed through open surgery (OGJ) or, more recently, via laparoscopy (Lap-GJ), which has demonstrated advantages in terms of reduced perioperative morbidity and mortality, less intraoperative bleeding, faster resumption of oral intake, and lower postoperative opioid requirements.^15^ Another option is antrectomy with or without vagotomy, particularly indicated in patients with refractory or recurrent peptic ulcer-related GOO. The reconstruction may vary among Billroth I, Billroth II, or Roux-en-Y.^16,17^ In selected patients with partial obstruction, pyloroplasty was also considered. The most commonly adopted techniques are Heineke-Mikulicz and Finney pyloroplasty, which aim to widen the pyloric channel and restore gastric emptying.^18^ While surgery offers a durable and often definitive solution, its clinical impact is not negligible. Postoperative complications may include surgical site infections (15%), anastomotic leak (1%), delayed gastric emptying (gastroparesis) (15%), and, in some cases, afferent loop syndrome or dumping syndrome (approximately 20% of patients undergoing vagotomy with pyloroplasty; up to 40% of patients after Roux-en-Y gastric bypass).^19–22^ These complications are especially relevant in frail or elderly patients, in whom recovery time may be significantly prolonged. In light of these considerations, surgical treatment for bGOO should be reserved for those cases where long-term benefit outweighs potential risks. Moreover, recurrent obstruction after surgical gastrojejunostomy is a rare condition, but it can be caused by certain chronic diseases, such as EoGE. In such cases, EUS-GJ may serve as a useful rescue therapy, avoiding another surgery since the underlying disease has reduced the durability of the SGJ.^23^ However, limited data are available so far to consider it a durable approach.

### Endoscopic balloon dilation

EBD is one of the available endoscopic treatment options for managing bGOO. First introduced in the early ‘80s, its clinical outcomes have progressively improved thanks to the refinement of techniques and the integration of targeted etiologic therapies. The procedure consists of the gradual expansion of the stenotic lumen using dilating balloons, with the aim of restoring gastric transit and alleviating symptoms. A prospective study published in 2007 by Cherian et al. described the experience in 23 patients with peptic-related GOO treated with EBD and etiological therapy (*H. pylori* eradication, NSAID withdrawal). With a median follow-up of 43 months, all patients achieved durable symptomatic remission, and 22 out of 23 reached endoscopic resolution (95.7%). In six patients (26.1%), long-term antisecretory therapy could be discontinued.^24^ These results were later confirmed and expanded by Kochhar et al., who evaluated 306 patients with bGOO, including 264 treated with EBD. In this larger cohort, clinical success was achieved in 92% of cases and procedural success in 75.7%, with an average of 5.3 sessions per patient. Response to treatment was highly dependent on etiology: caustic-induced GOO had the poorest outcomes (41.5% of refractory strictures), whereas non-caustic-related bGOO, including peptic ulcer-related and medication-induced strictures, showed excellent response rates, with refractory strictures (defined as the inability to dilate a stricture to a diameter of 15 mm after five sessions of EBD) in only 1.6% of cases.^25^ Complications such as perforation (3.4%), pain (9.1%), and bleeding (7.7%) were reported but were generally manageable. Minor adverse events (AEs) were treated conservatively, while severe AEs, including perforations or significant bleeding, were successfully managed either endoscopically or through prompt surgical intervention.^25,26^ Other studies, including those by Zare et al.^27^ and Hamzaoui et al.,^28^ have also supported the effectiveness of EBD in peptic GOO, while highlighting variability in long-term outcomes and the potential need for surgical intervention. Hamzaoui, in particular, reported a recurrence rate of 39.5%, with 29% of patients eventually requiring surgery, despite high initial efficacy.^28^ These findings are consistent with a systematic review including 14 retrospective studies and 596 patients, which concluded that EBD is particularly effective in peptic-related strictures, especially when associated with *H. pylori* eradication and NSAID cessation. Conversely, corrosive and pancreatitis-related strictures show lower responsiveness and often require multiple sessions.^29^ Therefore, outcomes and the number of sessions required vary significantly depending on the underlying cause. For peptic ulcer disease, EBD is highly successful, with clinical remission rates between 77% and 91%, and most patients achieving sustained relief after just two to three sessions; for example, one study found that 73% of patients improved after two sessions and did not require further dilation, while only about 9%–23% ultimately needed surgery.^25,30^ In contrast, caustic-induced obstructions are more challenging, requiring a higher average number of sessions (mean 7.25 vs 3.37 of non-caustic strictures) to achieve clinical success, which is lower compared to non-caustic strictures (90.1% vs 95.1%, *p* < 0.01),^25^ with most of the patients potentially needing surgery due to the high rate of recurrence (41.5% of caustic strictures vs 1.6%, of non-caustic, *p* < 0.01).^31,32^ Chronic pancreatitis-related obstructions respond poorly to EBD, despite the number of sessions being generally higher than other etiologies (mean of 5.5 sessions), with up to half of patients requiring surgery and the remainder often experiencing symptom recurrence.^30,31^ The above-mentioned study^29^ highlighted that strictures requiring repeated dilations are less likely to achieve durable resolution and may ultimately necessitate surgery. Repeated recurrence of stricture after EBD may be an indication for surgery. Overall, if more than two sessions of dilations are required, they are highly associated with the probability of surgery or alternative interventions.^33^ EBD is generally a safe procedure, with rare complications of bleeding and perforation in balloon diameters less than 15 mm. Perforation occurred more often when the diameter was over 15 mm.^34–37^ Overall, EBD represents an effective and minimally invasive treatment, particularly for uncomplicated peptic strictures. However, the high variability in response based on etiology, the potential need for repeated sessions, and the presence of complications underscore the importance of careful patient selection and prolonged clinical follow-up. A summary of the main studies evaluating EBD for benign GOO is presented in Table 2.

**Table 2.** Studies on endoscopic balloon dilation for benign gastric outlet obstruction.

Study	Year	Study design	No. Pts	Etiology	Mean Follow-up	Clinical success	Technical success	Complications	Mean No. sessions
Cherian et al.^24^	2007	Retrospective	23	100% Peptic ulcer (*H. pylori*, NSAIDs, idiopathic)	43 months	100%	NR	None	NR
Kochhar et al.^25^	2018	Retrospective	264	Mixed: caustic (53.78%), PUD (26.1%), drug-induced (22 pts, 8.3%), Tuberculosis (4 pts, 1.5%), Crohn’s disease (3 pts, 1.1%), Chronic pancreatitis (4 pts, 1.5%), other systemic diseases (20 pts, 7.57%)	78.6 ± 67.79 months	92.0% (overall)	75.7% (overall)	3.4% (9 perforations) 7.7% (20 bleeding) 9.1 (26 pain)	5.4 (clinical); 2.6 (technical)
Zare et al.^27^	2019	Retrospective	22	100% PUD	25.2 ± 10.3 months	73% after 2 sessions; 100% final	100%	None	2.2 (range 1–7)
Hamzaoui et al.^28^	2015	Retrospective	45	100% PUD	32 months	84.4% initial; 55.8% at 30 months	95.5%	4.5% (2 perforations) 2.2% (1 bleeding)	1.4 (range 1–4)
Boylan and Gradzka^34^	1999	Retrospective	40	100% PUD	⩾24 months	30% (no recurrence)	NR	NR	Multiple (not specified)
Chiu et al.^38^	2013	Retrospective	7	100% Caustic	30 ± 15.8 months	57.1%	NR	42.9%	5.5 ± 2.1
DiSario et al.^35^	1994	Retrospective	30	100% PUD	15 months (range 4–28)	80% (sustained relief)	86.6% (4 failures)	6.7% (2 perforations)	1.7 for patient
Hewitt et al.^39^	1999	Retrospective	46	100% PUD	19 months (range 1–78)	68% (non-surgical management)	89.1%	7.3% (3 perforations) 2.4% (1 bleeding)	2 (range 1–9)
Kochhar et al.^40^	2009	Retrospective	41	100% caustic	35.4 ± 11.1 months (range 18–58 months)	95.1%	100%	2.4% (1 perforation) 2.4% (1 surgery) 19.5% (8 pain) 17.1% (7 bleeding)	5.8 ± 2.6 (range 2–13)
Kochhar et al.^30^	2004	Retrospective	23	47.8% (11 peptic) 34.8% (8 caustic) 17.39% (4 Chronic pancreatitis)	Peptic: 14.0 ± 9.8 months; Caustic: 12–30 months	Good in peptic and corrosive; failure in pancreatitis	NR	NR	Peptic: 2.0 ± 0.63; Caustic: 5.63 ± 2.88; Pancreatitis: 5.5 ± 0.58
Lam et al.^36^	2004	Prospective	33	100% Peptic (14/25 H. pylori+, 11/25 H. pylori)	Median 24 months (IQR 16–40)	75.8% (25/33)	NR	27.3% (9 ulcer complications)	NR
Lau et al.^41^	1996	Retrospective	41	100% Pyloric stenosis	Median 39 months	83.3% initially; 51.2% needed surgery later	90.7%	7.4% (4 perforations) 2.4% (1 bleeding)	NR
Noor et al.^12^	2011	Retrospective	10	100% NSAID-induced pyloroduodenal obstruction	Median 12 months (IQR 2–16)	90%	100%	0%	2.0 (±1.6) to target; 5.3 (±2.7) total
Rana et al.^31^	2011	Retrospective	25	44% (*n* = 11) Peptic 28% (*n* = 7) caustic 16% (*n* = 4) Pancreatitis 8% (*n* = 2) TB 4% (*n* = 1) Crohn	Not reported	84% (21/25)	100% (assumed)	8% (2 perforations)	2.2 ± 1.2 (total) (caustic: 3.83 ± 0.75) (peptic: 2.1 ± 0.56)

NR, not reported; NSAIDs, nonsteroidal anti-inflammatory drugs; pts, patients; PUD, peptic ulcer disease; TB, tuberculosis.

### Self-expandable metal stents

The use of self-expandable metal stents (SEMS) in the management of bGOO remains a debated therapeutic option, generally reserved for patients who are not candidates for surgery or those with strictures refractory to EBD. Although SEMS placement can result in rapid symptomatic improvement, especially in cases of peptic obstruction, long-term outcomes and the safety profile appear less favorable compared to other treatment options. One of the main concerns is the risk of stent migration, which is particularly high with fully covered SEMS (FC-SEMS). These stents, although preferable for their removability and lower risk of in-stent hyperplasia, show migration rates as high as 28%. On the other hand, uncovered SEMS provide better anchorage to the wall but are more prone to occlusion due to reactive granulation tissue and tissue ingrowth. To reduce the risk of displacement with FC-SEMS, endoscopic fixation techniques such as stent suturing to the mucosa have been proposed.^42,43^ A recent prospective study including 22 consecutive patients with bGOO treated with FC-SEMS showed a technical success in 100% of cases, with a clinical success rate of 81.8% at 3 days and sustained improvement in 66.7% of patients at 8 weeks. However, stent migration occurred in 68.2% of cases, highlighting this complication as a major limitation to the broader use of FC-SEMS in this setting.^44^ Supporting these findings, a recent U.S. multicenter retrospective series evaluated the efficacy of retrievable FC-SEMS in patients with refractory benign strictures of the pylorus, duodenum, and gastrojejunal anastomosis. Stent placement followed by scheduled removal led to significant clinical improvement within 4 weeks, with a favorable tolerance profile and reduced need for additional interventions.^45^ Partially covered SEMS (PC-SEMS) have also been explored as a potential alternative. In a retrospective study of 10 patients with peptic GOO, PC-SEMS placement resulted in symptomatic improvement in all patients within 3 days, with a 90% durable remission rate at 11 months. This approach also proved valuable as a salvage therapy: among five patients who had previously failed endoscopic dilation, four experienced sustained clinical benefit. However, two cases of migration (20%) were recorded, both in patients with prior dilation.^46^ Based on the current literature, FC-SEMS may be considered a second-line treatment strategy following endoscopic dilation failure or as a temporary option in patients unsuitable for more definitive approaches such as surgery or EUS-GJ. Nevertheless, their use should be individualized and supported by a multidisciplinary team, with careful assessment of the expected benefits and potential risks. A summary of the main studies evaluating SEMS for benign GOO is presented in Table 3. However, an alternative GI stenting approach for short strictures (e.g., pyloric or anastomotic) involved the use of a lumen-apposing metal stent (LAMS), a double-flanged, self-expandable metal stent originally designed for EUS-guided procedures, which can be placed under direct endoscopic visualization through the stricture, similarly to a SEMS, in this alternative technique. The placement of the LAMS through the stricture showed high technical success across all gastrointestinal stricture sites (between 96.7% and 100%).^47–51^ A meta-analysis of 18 studies (527 patients) showed a 99.9% of technical success, even if it included heterogeneous benign strictures, as colonic and esophagogastric.^52^ Clinical success varied, with short-term symptom resolution in 93.9% of cases,^52^ with one large single-center study reporting 98.4%.^47^ For pyloric strictures specifically, pooled clinical success was 77.6% in a meta-analysis of six studies.^53^ Long-term clinical success, meaning sustained symptom relief after stent removal, was lower, reported at 66.7%,^50^ 72.8%,^54^ and 45%.^48^ AEs ranged from 13.3%^51^ to 30.6%,^53^ with stent migration rate being the most frequent complication, between 7% and 27.3%,^47,51^ followed by ulceration, bleeding, and perforation.^55^ Reintervention rate after LAMS removal is substantial, reported at 23% in pooled analysis,^52^ and 31.1% (even if it raised to almost 80% at 200 days of follow-up),^49^ 58.3%–83% for anastomotic stricture and 33%–50% for non-anastomotic stricture.^47,48^ Overall, LAMS have a limited long-term durability for this approach due to recurrence and the need for further intervention.

**Table 3.** Summary of studies using SEMS for bGOO.

Author	Year	Study design	Patients (*n*)	Stent type	Technical success	Clinical success	Follow-up	Complications
Choi et al.^44^	2013	Prospective	22	FC-SEMS	100%	81.8% early, 66.7% sustained	Mean 10.2 months	Migration 68.2%
Randhawa et al.^45^	2018	Retrospective	28	FC-SEMS	100%	100% at 4 weeks	Variable	Migration 28.6%
Heo et al.^46^	2014	Retrospective	10	PC-SEMS	100%	90% at median 11 months	Median 11 months (range 4–43)	Migration 20%

bGOO, benign gastric outlet obstruction; FC-SEMS, fully covered self-expandable metal stent; PC-SEMS, partially covered self-expandable metal stent.

### Endoscopic ultrasound-guided gastrojejunostomy

In recent years, EUS-GJ has emerged as an innovative and minimally invasive technique for the treatment of GOO. Initially proposed for malignant obstructions, it has also been extended to benign etiologies. The procedure involves the creation of an anastomosis between the stomach and the proximal jejunum under EUS guidance, typically using a LAMS. Interest in the application of EUS-GJ for benign GOO has grown in parallel with the experience gained in malignant settings and the limitations observed with other therapeutic approaches, such as EBD and placement of SEMS. LAMS offers a stable bypass route, theoretically more similar to a surgical anastomosis, with the advantage of avoiding surgery. Notably, one study reported the creation of an EUS-guided GJ in a benign setting using a fully covered tubular self-expandable metallic stent,^56^ although only two patients were included and one of them experienced asymptomatic stent migration, so the application of these stents for creation of EUS-GJ seems overcome in the era of LAMS, even if it could reduce costs. An important confirmation of the long-term efficacy of EUS-GJ using LAMS comes from a recent retrospective single-center study including 207 patients, 43.5% of whom were treated for bGOO. The study reported a technical success rate of 95.7% and a clinical success rate of 97.2%, with a median time to resumption of oral intake of just 1 day. Long-term AEs occurred in only 3.4% of cases and mainly included food impaction, gastrocolic fistulas, and stent lining degradation (delamination). In this setting, an annual scheduled stent exchange has been proposed for patients requiring prolonged stent indwelling to prevent mechanical complications associated with device permanence.^57^ Rizzo et al., in a recent meta-analysis, analyzed 15 studies including a total of 376 patients undergoing EUS-GJ for bGOO. The analysis reported a pooled technical success rate of 95.8% and a clinical success rate of 93.4%. The overall AEs rate was 11.6%, while symptomatic recurrence was observed in 11.6% of cases.^7^ These findings further support the efficacy and safety of EUS-GJ in benign indications, reinforcing its role as a valuable therapeutic option. This study also showed a weighted mean for stent removal time of 136.16 days among five studies,^58,59^ despite no specific indication can be given on the time of removal in bGOO based on data available. Nonetheless, Abel et al. noted that most of the cases in which they removed the LAMS and resolved the obstruction had an extrinsic cause of bGOO (three of four cases, 75%), so the reversibility of bGOO of these patients, given an appropriate time interval, permitted to safely remove the LAMS.^60^ A summary of the main studies evaluating EUS-GJ for bGOO is presented in Table 4. Although current data remain limited, EUS-GJ is now considered a promising therapeutic alternative in selected patients with bGOO, particularly in cases of complex, multifocal, or recurrent strictures after conservative treatment. The availability of experienced operators and structured multidisciplinary support are essential prerequisites for the safe and effective implementation of this technique.

**Table 4.** Summary of studies on EUS-GJ for bGOO.

First author, year	Design	Patients with bGOO, *n*	Eziology	Technical success, %	Clinical success, %	Adverse events, %	Follow-up
Khashab, 2015^61^	Retrospective, bicenter	7	Chronic pancreatitis: 3; NSAID stricture: 1; Peptic stricture: 1; Duodenal Hematoma: 1; SMAS: 1	90%	100%	0%	Mean 150 days
Chen, 2018^62^	Retrospective, multicenter	26	Chronic pancreatitis: 11; Surgical anastomosis: 6; Peptic ulcer disease: 5; Acute pancreatitis: 1; SMAS: 1.	96.2 %	84%	11.5% (3/26: 2 misdeployments, 1 gastric leak post elective stent removal)	Median 176.5 days (IQR 47–445.75)
Kerdsirichairat, 2019^63^	Retrospective, bicenter	9	Peptic stricture (*n* = 2); Surgical anastomotic stricture (*n* = 2); SMAS (*n* = 1); Small bowel Crohn’s disease (*n* = 1); Severe adhesion (*n* = 1); Severe intramural duodenal hematoma from acute pancreatitis (*n* = 1); Caustic injury (*n* = 1)	93%	89.5%	3.5% (2/57 overall: 1 severe perforation, 1 moderate)	Median 319.5 days
James, 2020^59^	Retrospective, single center	22	Peptic stricture (*n* = 5), Anastomotic stricture (*n* = 4); Duodenal hematoma (*n* = 3); Recurrent acute pancreatitis (*n* = 1); Chronic pancreatitis (*n* = 4); Pancreatic pseudocyst (*n* = 1) and walled off pancreatic necrosis (*n* = 4).	95.5%	83.3%	13.6%	Mean 564 days
Sameera, 2020^64^	Retrospective, single center	17	Groove pancreatitis (*n* = 14); SMA syndrome (*n* = 1); postsurgical inflammation (*n* = 1); Other 1	93%–95%	90%–92%	NR	NR
Sobani, 2021^65^	Retrospective, single center	8	Peptic stricture (*n* = 4); chronic pancreatitis (*n* = 2); SMA syndrome (*n* = 2)	100%	93.5%	3.23%	Mean 291.75 ± 211.40 days
Havre, 2021^66^	Retrospective, bicenter	5	Aorto-duodenal syndrome (*n* = 2); Chronic pancreatitis (*n* = 3)	100%	100%	20% (1/5, late stent migration)	Median 26 (20–135) weeks
Kahaleh, 2023^67^	Retrospective, multicenter	31	Stricture from various causes	100%	10%	NR	Mean 6.4 months
Rizvi, 2023^68^	Retrospective, multicenter	72	Surgical anastomotic stricture (*n* = 14; 19%); Peptic stricture (*n* = 12; 17%); Chronic pancreatitis (*n* = 11; 15%); Acute pancreatitis (*n* = 9; 13%); SMAS (*n* = 7; 10%); Caustic stricture (*n* = 4; 6%); Crohn’s disease stricture (*n* = 2; 3%); Duodenal hematoma (*n* = 2; 3%)	97%	71% on full diet; symptom improvement 90% at 60 days	6% procedural AEs	Mean 426.3 (±443.9) days
Abel, 2024^60^	Retrospective, single center	18	Pancreatitis (*n* = 6); NSAID-induced stricture (*n* = 4); Peptic stricture (*n* = 2); SMA syndrome (*n* = 3) radiation-induced stricture (*n* = 1); Anastomotic stricture (*n* = 1) duodenal hematoma (*n* = 1)	100%	94%	0%	NR
Fimiano, 2024^69^	Retrospective, single center	8	Acute pancreatitis (*n* = 2); Groove pancreatitis (*n* = 1); Chronic pancreatitis (*n* = 3); Postsurgical stricture (*n* = 1); Peptic disease (*n* = 1)	100%	100%	12.5%	Mean 12 (±6) months
Martinez Ortega, 2024^70^	Retrospective, multicenter	69	Intraluminal GI stricture (11 peptic, 3 anastomotic, 1 other)	97.1%	89.6%	10.1% (3 mild fevers, 1 severe bleeding, 1 fatal bleeding)	Median 1.2 years (IQR 0.33–1.3)
Wannhoff, 2024^58^	Retrospective, multicenter	39	Biliary pancreatitis (*n* = 14) = Alcoholic pancreatitis (*n* = 13) = Post-ERCP pancreatitis (*n* = 3) = Pancreatitis - other (*n* = 8)	92.3%	87.2%	2.6% (1 gastrocolic fistula)	Median 23 months (range 3–45)°
Gonzalez, 2024^71^	Retrospective, single center	34	Chronic pancreatitis (9); gastroparesis (16), Aorto-mesenteric pince (1), Crohn (2), caustic (1), surgical dysfunction (2)	94.1%	94.1%	17.6%	Mean 27.76 (±94.46) months
Şentürk, 2024^56^	Retrospective, single center	2	Duodenal stricture due to peptic ulcer (1); early obstruction of the gastrojejunostomy site after Whipple procedure for IPMN (1)	100%	100%	50% (1 stent migration)	NR
Rizzo, 2025^72^	Retrospective, multicenter	11	Chronic pancreatitis stricture (6), pyloric stricture after chemiotherapy (2), anastomotic stricture (1), petic stricture (1), fluid collection after surgery causing compression (1)	90.9%	70%	18.2%	Median 200 (IQR 482) days
Majumder et al., 2016^73^	Retrospective single-center	5	Gastroduodenal strictures, gastrojejunostomy stenosis	100%	100%	0%	Short-term (up to 3 months post-procedure; some stents left in place indefinitely)
Trieu et al., 2025^57^	Retrospective single-center study	90	Mixed benign etiologies	95.7%	97.2%	3.4% (long-term AEs)	Long-term

AEs, adverse events; EUS, endoscopic ultrasound; GI, gastrointestinal; IPMN, intraductal papillary mucinous neoplasia; IQR, interquartile range; LAMS, lumen-apposing metal stent; NR, not reported; NSAID, nonsteroidal anti-inflammatory drugs; SD, standard deviation; SMAS, superior mesenteric artery syndrome.

## Comparative studies on the treatment of bGOO

### Endoscopic ultrasound-guided gastrojejunostomy versus enteral stenting

In recent years, several comparative studies have been published evaluating the main techniques used in the management of bGOO. Most comparative studies between EUS-GJ and SEMS have predominantly involved patients with malignant GOO so far, even if several important observations can be adopted to benign conditions from studies with mixed etiology. In an early retrospective study by Iqbal et al., including 60 patients (8% with bGOO), EUS-GJ and SEMS showed comparable technical success (100%) and similar clinical success rates (87.5% vs 90.4%, respectively). However, EUS-GJ was associated with a lower rate of symptom recurrence and need for reintervention compared to SEMS. Specifically, no reinterventions were required in the EUS-GJ group, whereas 17% of patients in the SEMS group experienced recurrence requiring further treatment, with a median time to reintervention of 94 days (IQR: 43–112). These findings highlight the potential benefit of EUS-GJ in terms of long-term symptom control and reduced need for additional procedures, even in the context of bGOO.^74^ More recently, a retrospective multicenter study by Dzwonkowski et al. evaluated 103 patients with GOO, of whom approximately 40.9% in the EUS-GJ group and 11.1% in the ES group had benign disease. While technical success was 100% for both approaches and clinical success rates were comparable (97.7% for EUS-GJ vs 90.5% for SEMS, *p* = 0.24), EUS-GJ was significantly associated with fewer unplanned reinterventions. In a multivariate analysis adjusted for etiology (benign vs malignant), EUS-GJ independently reduced the risk of reintervention (HR: 0.264; 95% CI: 0.086–0.813; *p* = 0.02), although separate subgroup outcomes for bGOO were not specifically reported.^75^ The largest available comparative study, conducted by Jaruvongvanich et al., included 436 patients (approximately 17% with bGOO) treated with EUS-GJ (*n* = 232), SEMS (*n* = 131), or surgery (*n* = 73). EUS-GJ demonstrated a significantly lower reintervention rate (0.9% vs 12.2% for SEMS and 13.7% for surgery, *p* < 0.0001) and a lower AEs rate (8.6% vs 38.9% vs 27.4%, respectively). Clinical success was also superior with EUS-GJ (98.3%) compared to SEMS (91.6%) and surgery (90.4%).^76^ Although subgroup analyses focusing exclusively on b GOO were not reported, these results suggest the use of EUS-GJ as a preferred approach, offering more durable outcomes and fewer complications than SEMS.

### Endoscopic ultrasound-guided gastrojejunostomy versus surgical gastrojejunostomy

Over the past few years, multiple retrospective studies have compared EUS-GJ and SGE approaches in the management of GOO, including benign cases. An international multicenter study compared EUS-GJ and Lap-GJ in 54 patients with GOO, showing a favorable trend toward the endoscopic approach in terms of safety. Although the technical success rate was slightly lower for EUS-GJ compared to Lap-GJ (88% vs 100%; *p* = 0.11), the clinical success rate was similar (84% vs 90%; *p* = 0.11). The incidence of AEs was significantly lower in the EUS-GJ group compared to the Lap-GJ group (12% vs 41%; *p* = 0.038), suggesting potentially greater safety of the endoscopic approach, even in a cohort of more clinically complex and compromised patients. However, the study did not report a separate analysis for benign etiologies, limiting the direct applicability of the results to this subgroup.^77^ More robust data emerged from a 2024 retrospective multicenter study that directly compared EUS-GJ and SGE in 97 patients, of whom 36.1% had bGOO. This analysis demonstrated comparable technical success (*p* = 0.133), clinical success (*p* = 0.229), severe morbidity (*p* = 0.708), 30-day mortality (*p* = 0.277), and GOO recurrence rates (*p* = 1.000) between the two techniques. However, EUS-GJ was significantly associated with shorter procedural time, lower incidence of post-procedural ileus, and reduced hospital stay (all *p* < 0.001). In the bGOO subgroup, SGJ achieved a slightly higher technical success rate (*p* = 0.035), likely reflecting the anatomical complexity and higher technical demands of the endoscopic procedure. Nevertheless, once technically successful, both approaches provided similar rates of long-term symptom relief.^78^ Further support for the endoscopic approach comes from a single-center study comparing EUS-GJ and OGJ in 66 patients. While technical and clinical success rates were similar between groups, EUS-GJ offered clear advantages in terms of faster return to oral intake (1.3 vs 4.7 days), shorter hospitalization (5 vs 14.5 days), and significantly lower costs ($49,387 vs $124,192), even if the latter data are dependable by the country and regional health system. Although the majority of cases were likely malignant, these findings further highlight the practical and economic benefits of EUS-GJ even in complex clinical settings.^79^ Finally, a European multicenter study using propensity score matching compared Lap-GJ and EUS-GJ in a matched cohort of 74 patients, 5.4% of whom had benign GOO. Rates of technical and clinical success were comparable (94.6% vs 100% and 97.1% vs 89.2%, respectively), but EUS-GJ proved superior regarding time to resume oral intake (median 1 vs 3 days), time to full diet (2 vs 9 days), and length of hospital stay (4 vs 8 days). Furthermore, the endoscopic approach was associated with significantly fewer overall and serious AEs.^80^ Therefore, the advantages of surgery in benign diseases include a higher probability of technical success, as it is a relatively simple procedure and offers a more “permanent” solution. In contrast, EUS-GJ requires greater expertise and training to achieve a similar technical success rate, even if nowadays it is spreading fast. Moreover, the recurrence rate and the need for endoscopic revisions indicate the lower durability of the GJ fistula. Nonetheless, long-term data are needed to better explore this issue. On the other hand, surgery carries the disadvantage of potential complications, both open and laparoscopic, and typically results in a longer hospital stay, which negatively impacts healthcare costs. Conversely, the EUS-guided approach helps reduce hospital stay and the risk of AEs. A summary of comparative studies on EUS-GJ versus other treatments for benign is presented in Table 5.

**Table 5.** Comparative studies on EUS-GJ versus other treatments for bGOO.

Study	Design	Comparison	No. patients with bGOO	Technical success	Clinical success	Recurrence	Reinterventions	Adverse events
Jaruvongvanich et al. 2022^76^	Retrospective multicenter	EUS-GJ vs ES vs Surgical GE	436 pts (17% benign, 74/436)	98.3% vs 99.2% vs 100% *p* = 0.58	98.3% vs 91.6% vs 90.4% *p* < 0.0001	NR	0.9% vs 12.2% vs 13.7% *p* < 0.0001	8.6% vs 38.9% vs 27.4% *p* < 0.0001
Dzwonkowski et al. 2022^75^	Retrospective single-center study	EUS-GJ vs ES	103 pts 11.1% benign in ES group, 40.9% benign in EUS-GE group	100% vs 100%	90.5% 97.7% *p* = 0.24	NR	9.1% vs 20.6% *p* = 0.11	6.8% vs 3.2% *p* = 0.4
Iqbal et al. 2019^74^	Retrospective single-center	EUS-GJ vs ES	60 pts (8% benign GOO)	100% vs 100%	87.5% vs 90.4% *p* = 1.00	NR	0% vs 17%	12.5% vs 3.8% *p* = 0.35
Martinet et al. 2024^78^	Retrospective multicenter	EUS-GJ vs SGE	97 pts total (36.1% benign)	Comparable overall (*p* = 0.133); higher for surgery in benign cases (*p* = 0.035)	Comparable between groups (*p* = 0.229 overall; *p* = 1.000 in benign subgroup)	Comparable between groups (*p* = 1.000)	Not separately reported; assumed similar based on recurrence data	Lower rate of post-procedural ileus with EUS-GJ (*p* < 0.001); severe morbidity comparable (*p* = 0.708)
Kouanda et al. 2021^79^	Retrospective single-center	EUS-GJ vs OGJ	66 patients (etiology mixed)	92.5% EUS-GE vs 100% OGJ (*p* = 0.15)	Comparable between groups. EUS-GE had faster oral intake (1.3 vs 4.7 days, *p* < 0.001), shorter stay (5 vs 14.5 days, *p* < 0.001), lower costs ($49,387 vs $124,192, *p* < 0.001)	17.5% EUS-GE vs 19.2% OGJ (*p* = 0.34)	20% EUS-GE vs 11.5% OGJ (*p* = 0.78)	No major difference;
Bronswijk et al. 2021^80^	Retrospective international multicenter study	EUS-GJ vs Lap-GJ	74 matched patients (5.4% with benign GOO)	94.6% vs 100% *p* = 0.493	97.1% vs 89.2% *p* = 0.358	No recurrence (GE dysfunction) in either group	Surgical reintervention needed in 3 Lap-GJ patients (8.1%)	2.7% vs 27% *p* = 0.007; Severe AE 0% vs 16.2% *p* = 0.025

AE, adverse event; bGOO, benign gastric outlet obstruction; EBD, endoscopic balloon dilation; ES, enteral stenting; EUS-GE, endoscopic ultrasound-guided gastroenteroanastomosis; EUS-GJ, endoscopic ultrasound-guided gastrojejunostomy; Lap-GJ, laparoscopic gastrojejunostomy; OGJ, open gastrojejunostomy; SEMS, self-expandable metal stent; SGE, surgical gastrojejunostomy.

## Discussion

The management of bGOO has undergone significant evolution in recent years, driven by the development of advanced endoscopic techniques. Historically, SGJ has been considered the standard treatment, particularly in young and otherwise healthy patients, offering long-lasting symptomatic control. However, this approach is associated with non-negligible morbidity, especially in elderly or frail patients, and requires a prolonged postoperative recovery.^2^ EBD has established itself as a minimally invasive alternative, particularly effective in peptic strictures. However, its efficacy significantly decreases in non-peptic forms (e.g., chronic pancreatitis and postsurgical strictures), where anatomical distortion or fibrosis compromises long-term success. Repeated sessions are often necessary, and recurrence rates can be high.^29^ In this context, EUS-GJ has proven to be a promising technique using LAMS, capable of combining the physiological efficacy of surgical bypass with the minimally invasive nature of the endoscopic approach. Several retrospective studies and a recent dedicated meta-analysis have shown high technical and clinical success rates (up to 98% and 96%, respectively), with relatively low rates of recurrence and complications.^7^ These data suggest that EUS-GJ could represent a first-line option in selected patients with b GOO, particularly in complex, multifactorial, or recurrent cases.

However, EUS-GJ still presents some limitations. Most of the available evidence comes from retrospective studies with short- to medium-term follow-up, and solid data on long-term efficacy are lacking, which is fundamental in a context of benign diseases. Therefore, the timing of LAMS removal is not standardized, lacks specific guidelines, and many operators follow their internal protocols and clinical expertise to determine when to remove the LAMS or whether to replace it periodically. Consequently, future studies should incorporate this aspect into their protocols. In addition, the EUS-GJ technique requires a high level of expertise and access to dedicated devices, which are still off-label for this indication. It should also be emphasized that in young and fit patients, surgery often remains the preferred option, as it provides a definitive solution with durable efficacy and fewer risks of late stent-related complications. FC-SEMS may still be considered as a temporary option in non-operable patients or in refractory cases, but the risk of migration and the need for close follow-up limit their routine use. Li et al., in a recent comprehensive meta-analysis including 26 studies and 1493 patients with both bGOO and mGOO, confirmed that EUS-GJ achieves high technical (94.0%) and clinical (89.9%) success rates with an acceptable overall AEs rate (13.1%).^81^ Compared with SGJ, EUS-GJ was associated with similar efficacy but a significantly lower risk of AEs. When compared to ES, EUS-GE showed higher clinical success and a lower complication rate, supporting its role as an effective minimally invasive option for GOO, even in the context of mixed etiologies.^81^

## Conclusion

In conclusion, we suggest to manage bGOO in the context of a multidisciplinary team in tertiary centers, taking into account the patient’s clinical condition, age, surgical risk, and the etiology of obstruction for guiding therapeutic decision-making. Overall, EBD remains the first-line approach for peptic strictures, while EUS-GJ should be considered for non-peptic forms, recurrent peptic strictures, or anatomically complex cases, particularly in frail patients or those at high surgical risk. Surgery continues to have a central role in having a more definitive treatment, especially in young and fit patients or in cases where endoscopic approaches are not feasible or effective. FC-SEMS may have a role as a bridge-to-surgery in those cases who need to undergo elective surgery. Our study may help physicians and multidisciplinary boards in the decision-making process, providing a structured and evidence-informed framework. Future studies should focus on prospective comparisons with longer follow-up, better patient stratification (e.g., by etiology), and cost-effectiveness analyses to more precisely define the optimal therapeutic option in the treatment of bGOO.

## Declarations

**Ethics approval and consent to participate:** Not applicable.

**Consent for publication:** Not applicable.

**Availability of data and materials:** Not applicable.
